# The association between H. pylori infection and cognitive deterioration: a systematic review and meta-analysis

**DOI:** 10.1186/s40001-025-03160-8

**Published:** 2025-09-16

**Authors:** Mahmoud M. Elhady, Abdelrahman Zidan, Eslam Mohammed Rabea, Heidi Sherif Farouk, Moustafa Z. Elattar, Manar Adel, Mariam Abdulkhaliq Khalil, Amira A. Aboali, Marwa Abdel Aziz Zeid, Mohamed A. Shaltout, Alzahraa M. Abdel-Daim, Asmaa Gomaa Alwarraqi, Hazem AbuEl-Enien, Mohamed Sayed Zaazouee

**Affiliations:** 1 Faculty of Medicine, Benha University, Qalubiya, Egypt; 2 Emory Clinical Cardiovascular Research Institute, Emory University, Atlanta, GA 30322 USA; 3 Faculty of Medicine, Alexandria University, Alexandria, Egypt; 4 Anatomy & Embryology Department, Benha Faculty of Medicine, Benha University, Qalubiya, Egypt; 5 Faculty of Clinical Pharmacy, Tanta University, Gharbia, Egypt; 6 Damanhour Teaching Hospital, General Organization for Teaching Hospitals and Institutes, Damanhour, Egypt; 7 Faculty of Medicine, Suez Canal University - Public Health and Community Medicine Department, Ismailia, Egypt; 8 Faculty of Medicine, Al-Azhar University, Assiut, Egypt; 9 Postdoctoral Fellow, Section of Infectious Diseases, Department of Internal Medicine, Yale School of Medicine, New Haven, USA; 10 Faculty of Medicine, New Ismailia National University, Ismailia, Egypt; 11 Independent Researcher, Abu Dhabi, United Arab Emirates

**Keywords:** H. pylori, Dementia, Cognitive decline, Infection, Meta-analysis, Association

## Abstract

**Background:**

The association between cognitive decline and Helicobacter pylori (H. pylori) infection remains controversial, with some evidence suggesting that H. pylori eradication may slow the progression of the disease. This meta-analysis aims to investigate the bidirectional relationship between H. pylori and cognitive decline.

**Methods:**

We searched PubMed, Web of Science, the Cochrane Library, and Scopus for double-arm studies that reported either the prevalence of cognitive decline in individuals with H. pylori-positive status or the prevalence of H. pylori infection in patients with cognitive decline. A random-effects meta-analysis was conducted using Comprehensive Meta-Analysis software to pool the odds ratios from the included studies. Study quality was assessed using the Newcastle–Ottawa Scale.

**Results:**

Our search identified 1,240 records, with 16 studies meeting the inclusion criteria. Meta-analysis showed that patients with H. pylori had a significantly higher risk of cognitive decline (OR = 1.338, 95% CI 1.046–1.713), with the strongest association seen in studies grouping cognitive dysfunction and dementia (OR: 3.190, 95% CI 1.853–5.490). However, the risk of Alzheimer’s disease was insignificant. Cognitive decline cohorts showed a significantly higher prevalence of H. pylori (OR = 1.5, 95% CI 1.131–1.989), with a significant association with Alzheimer’s disease (OR: 1.630, 95% CI 1.064–2.497), but not with dementia or cognitive dysfunction. The association varied across study designs, with cross-sectional studies showing no association in both analyses. Heterogeneity was substantial (*I*^2^ > 70% in most analyses), highlighting variability in the findings.

**Conclusion:**

This meta-analysis indicates a bidirectional association between H. pylori and cognitive decline. While H. pylori infection increased the overall risk of cognitive decline, no significant link was found with Alzheimer's disease. Conversely, Alzheimer’s disease patients had a higher prevalence of H. pylori infection. High heterogeneity underscores the need for further well-designed studies to clarify this relationship.

**Supplementary Information:**

The online version contains supplementary material available at 10.1186/s40001-025-03160-8.

## Introduction

Cognitive decline is a broad term referring to a deterioration in thinking, memory, and reasoning abilities that can occur with aging or due to underlying conditions [1]. It spans a clinical continuum from mild cognitive impairment (MCI), which has limited impact on daily functioning, to dementia, a disabling condition that severely impairs independence and quality of life [2]. Dementia is a major and escalating public health challenge. Globally, over 55 million people Live with dementia, and nearly 10 million new cases are diagnosed each year, with projections estimating 152 million cases by 2050 [3]. Alzheimer’s disease (AD) accounts for 60–70% of cases, followed by vascular dementia, Lewy body dementia, and frontotemporal dementia [1, 4, 5]. The economic impact is also immense—global dementia-related healthcare costs exceeded $1.3 trillion USD in 2019 and are expected to double by 2030 [6].

In addition to aging, gender, and education level, several modifiable medical conditions—such as diabetes, cardiovascular disease, and stroke—are recognized contributors to cognitive decline [7–10]. Recently, infections have gained attention as potential risk factors in neurodegeneration, particularly AD [11, 12]. Emerging evidence has implicated various pathogens, including herpes simplex virus type 1 (HSV-1), Chlamydia pneumoniae, Helicobacter pylori, and even SARS-CoV-2, in the pathogenesis of cognitive impairment dementia [13–18]. These findings have prompted investigations into the possible links between chronic infection, systemic inflammation, and cognitive deterioration.

In this article, we focus on H. pylori due to its high global prevalence, estimated at 43.9% between 2015 and 2022 [19]. Its widespread distribution, particularly in developing countries, is primarily attributed to poor sanitation, overcrowding, and limited access to clean water [20]. The bacterium’s ability to persist in the gastric antrum—its preferred site—for years, often without causing symptoms, further contributes to its extensive prevalence [21]. On a clinical level, H. pylori infection has been linked to various conditions, including gastric and duodenal ulcers, gastric cancer, and mucosa-associated lymphoid tissue (MALT) lymphoma [22, 23]. In contrast, the possible connection between H. pylori infection and cognitive decline remains controversial and understudied.

Although some observational studies have proposed a possible link, the current body of evidence is inconclusive, with findings ranging from no association [24] to a statistically significant relationship [25–27]. This inconsistency may reflect methodological differences, population heterogeneity, or residual confounding. Several biological mechanisms have been proposed to explain how H. pylori infection may contribute to cognitive impairment. These include chronic systemic inflammation, which may exacerbate neuroinflammatory pathways, and molecular mimicry triggering autoimmune responses against neural tissues [28, 29]. Additionally, H. pylori-associated vitamin B12 and folate deficiencies may impair neuronal function, while vascular endothelial dysfunction may promote cerebrovascular disease [30]. Though these pathways remain hypothetical, they offer a rationale for exploring a potential association between infection and neurodegeneration. Given the uncertainty and growing interest in infection-related neurodegeneration, we conducted a systematic review and meta-analysis to synthesize available data and evaluate whether an association exists between H. pylori infection and cognitive impairment.

## Methods

This systematic review and meta-analysis was conducted following the guidelines outlined in the Preferred Reporting Items for Systematic Reviews and Meta-Analyses (PRISMA) statement [31].

### Search strategy and study selection

A comprehensive systematic literature review was conducted using PubMed, the Cochrane Library, Web of Science (WOS), and Scopus, covering all records from their inception until February 10, 2025. No restrictions or filters were applied. The search included the following terms: Dementia, Alzheimer, cognitive decline, cognitive dysfunction, cognitive impairment, mental deterioration, neurocognitive decline, neurocognitive dysfunction, neurocognitive impairment, neurocognitive deterioration, neurological decline, neurological deterioration, memory loss, memory disorder, memory dysfunction, Pylori, Campylobacter pylori, Helicobacter pylori, and H. pylori. Details of the search strategy and results are provided in Supplementary Table 1. Two independent reviewers assessed the records for relevance. Articles deemed relevant based on title and abstract screening underwent a detailed full-text evaluation. Any disagreements were resolved through consensus with a third reviewer.

### Eligibility criteria

Any study investigating the association between H. pylori and any form of dementia was included, provided it reported either the prevalence of H. pylori in dementia patients or the prevalence of dementia in individuals with H. pylori infection. Only studies with a comparative (double-armed) design were eligible. Eligible studies were required to report events and totals for each group or directly provide odds ratios (OR) or risk ratios (RR).

We excluded single-arm studies lacking a control or comparison group, as well as case reports and case series. Additionally, studies reporting effect estimates solely as hazard ratios (HRs) were excluded due to their incompatibility with meta-analytic pooling of ORs and RRs. We also excluded studies that did not provide sufficient data, such as missing sample sizes, number of events, or effect estimates with corresponding precision measures. Finally, non-English language articles were excluded unless a full English translation was available.

### Data extraction and quality assessment

After reaching a consensus on the final List of eligible studies, two researchers independently extracted relevant data from each study. The extracted data, including study summaries, baseline characteristics, and outcomes, were recorded in Excel sheets. Key variables collected included the year of publication, study location, study design, sample size, follow-up duration, dementia definition, H. pylori detection method, and primary outcomes. Additionally, odds ratios with their 95% confidence intervals were calculated or directly extracted and compiled for analysis.

To assess the quality of the included studies, the Newcastle–Ottawa Scale (NOS) was used. Three versions of the NOS were applied based on study design: one for cohort studies, one for case-control studies, and a modified, adapted version for cross-sectional studies, as used in previous systematic reviews [32, 33].

### Statistical analysis and subgrouping

The studies were classified into two groups: those assessing the odds of dementia in individuals with H. pylori infection and those examining the prevalence of H. pylori positivity among patients with dementia. Quantitative analysis was conducted using Comprehensive Meta-Analysis (CMA) software. Pooled odds ratios (ORs) with corresponding 95% confidence intervals (CIs) were derived from the included double-arm studies. A random-effects model was applied throughout the analysis. Heterogeneity is identified when *I*^2^ ≥ 50% and *p* ≤ 0.1). Heterogeneity was considered present when I^2^ ≥ 50% and *p* ≤ 0.10. The degree of heterogeneity was graded as follows: low (*I*^2^ = 0–25%), moderate (26–50%), substantial (51–75%), and considerable (> 75%) [34]. In cases of substantial or considerable heterogeneity, meta-regression was employed to identify contributing factors. Dementia is diagnosed clinically rather than through psychometric tests, requiring significant memory impairment along with at least one of the following: aphasia, agnosia, apraxia, or executive dysfunction [35]. The included studies varied in their disease definitions, encompassing impaired mental health, cognitive impairment, all-cause dementia, and AD. Studies that relied solely on psychometric tests were classified under the cognitive dysfunction subgroup, while those incorporating clinical assessments or explicitly identifying dementia were categorized as dementia. One study evaluated both groups together. Based on these definitions, we conducted subgroup analyses, along with an additional subgroup analysis based on study design. To assess potential publication bias, Egger’s test and funnel plots were utilized; an asymmetrical funnel plot or a statistically significant Egger’s test result would indicate the presence of publication bias [36].

## Results

### Search results

**Fig. 1:**
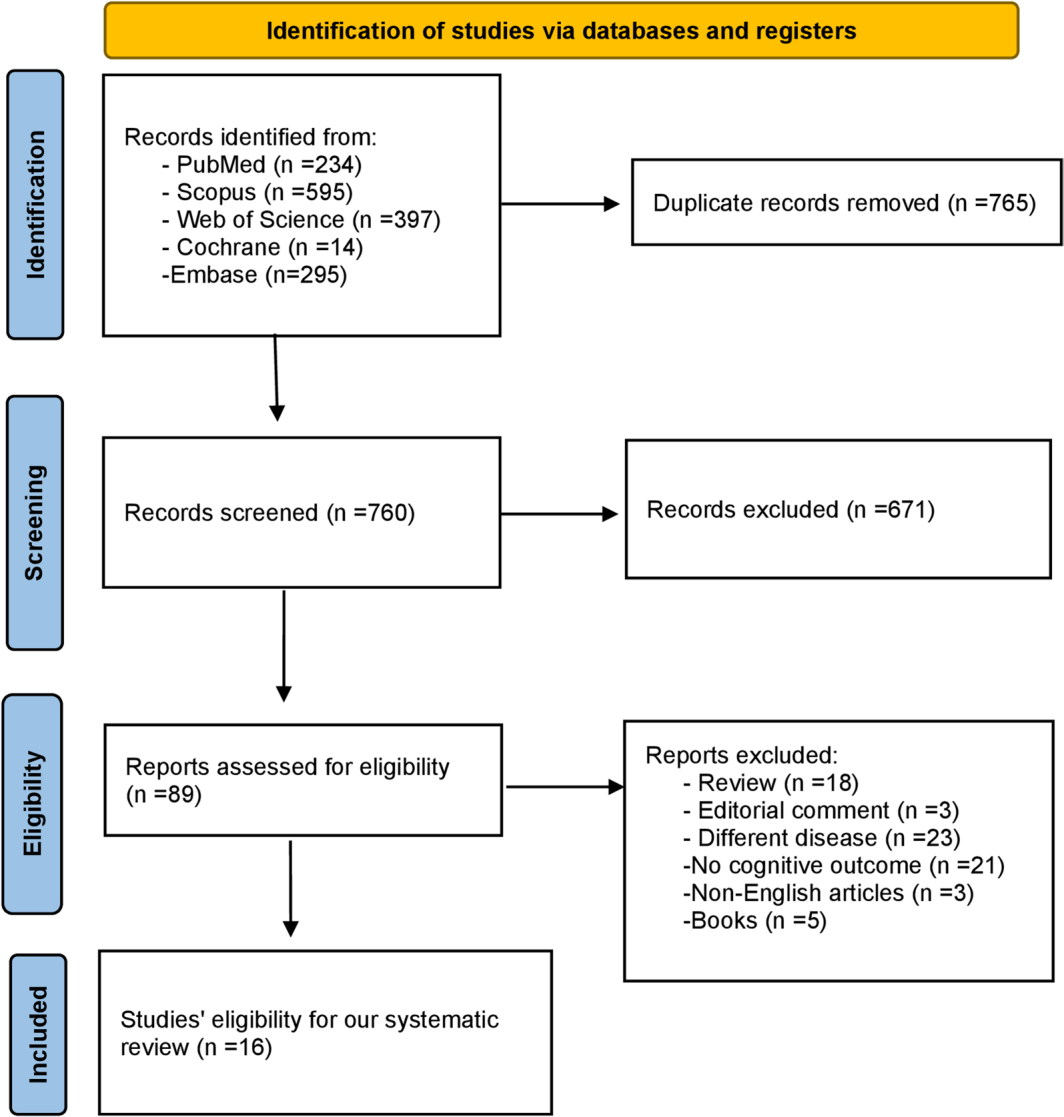
Prisma flowchart

Our search identified 1,240 records in total. After removing 575 duplicates, 665 studies remained for title and abstract screening, of which 84 were deemed relevant. Following a thorough Full-text review, only 16 studies met the inclusion criteria and were incorporated into the meta-analysis [24–26, 37–49]. The PRISMA flowchart is demonstrated in Fig. 1.

### Study characteristics

Among the 16 observational studies, 5 were cohort studies [26, 39, 40, 43, 47], 5 were cross-sectional [24, 44, 45, 48, 49], and 6 were case-control studies [25, 37, 38, 41, 42, 46]. Study populations ranged from small cohorts of 53 participants to large-scale datasets exceeding 1.6 million patients. The studies spanned multiple countries, including the United States, the United Kingdom, China, Taiwan, Japan, Greece, Iran, France, and Singapore, reflecting diverse geographical and healthcare settings. H. pylori detection methods varied, with most studies relying on serological tests (IgG ELISA or EIA), while others used histology, urease testing, gastroscopy, or rapid urine tests. Follow-up durations ranged from a few years to over a decade, with the longest being 14 years. Of the 16 included studies, 9 investigated cohorts of H. pylori-positive individuals to evaluate the subsequent incidence of dementia [24, 26, 39, 40, 43, 45, 47–49], while the remaining studies focused on patients with pre-existing dementia to assess the prevalence of H. pylori infection [25, 37, 38, 41, 42, 44, 46]. All studies employed a double-armed design with appropriately matched control groups free of the index disease. Summary and characteristics of the included studies are demonstrated in Tables 1 and 2.

**Table 1:** characteristics of the included studies

Study name	Study site	Study design	Total patients	Disease details in analysis	H. Pylori detection	Follow-up duration	Primary outcome
Beydoun 2018	US	Retrospective cohort	5,927	AD	IgG ELISA	Until December 31, 2011	Incidence of AD, all-cause dementia, and AD-related mortality
Beydoun 2020	US	Cross-sectional	1,439	AD	H. pylori IgG ELISA	Until 2014	AD and dementia incidence
Bu 2015	China	Case-control	263	AD	Serology (IgG antibodies)	Not specified	Cognitive impairment association with infectious burden (not H. pylori alone)
Cardenas 2019	US	Cross-sectional	2,894	CD	Serology (IgG EIA)	Data collected over two time periods (1988–1991, 1999–2000)	Cognitive impairment based on MMSE and DSST scores
Douros 2024	UK	Case-control	1,650,957	AD	Clinically apparent infection (CAHPI) based on testing and treatment records	Mean 11 years	Risk of AD associated with H. pylori
Fu 2024	UK	Longitudinal cohort study	8,144	Dementia	IgG antibodies (H. pylori VacA)	14.08 years	Risk of dementia/AD related to pathogen exposure
Han 2017	Taiwan	Cross-sectional	587	CD	Serum H. pylori IgG level measured using a commercial enzyme immunoassay (IMMULITE 2000, Siemens) |	2011–2013 (Health check program)	Association between H. Pylori IgG quartiles and cognitive impairment
Huang 2014	Taiwan	Retrospective cohort	83,965	Dementia	Diagnosis through ICD-9 code 041.86	13 year	Development of dementia (ICD-9 codes for dementia used)
Kountouras 2006	Greece	Prospective cohort	80	AD	Histology and Urease test	Not specified	Alzheimer's Disease linked to H. pylori infection
Koyama 2016	Japan	Case-control	151	CD	H. pylori antibodies using ELISA	8 years	Serum parameters with cognitive decline risk
Lu 2022	China	Prospective cohort	884	Impaired Mental Health	IgG antibody titters by ELISA	8 years	Total pathogen burden -Geriatric Depression Scale (GDS) score and Mental Component Score (MCS), Inflammatory markers
Rezaeimehr 2016	Iran	Cross-sectional	1,514	CD	IgG antibody titters against HP using ELISA	n/a	Examine this relationship between H. Pylori infection and cognitive impairment in Iranian elderly. Mini-mental-mental state examination (MMSE) score
Roubaud-Baudron 2012	France	Cross-sectional	53	AD	IgG H. pylori antibodies by ELISA and immunoblot	n/a	Cognitive impairment, neuroinflammation, and cerebrovascular lesion load in a group of AD patients according to their H. pylori status—we assessed: clinical data, biological data, and amyloid beta peptide levels, serum/CSF-cytokines and pepsinogen I/pepsinogen II (PgI/PgII) ratio, and cerebrovascular lesion load
Shi 2024	Singapore	Prospective cohort	885	CD + Dementia	ELISA to measure the IgG antibody titers	3–5 years	Seropositivity to 11 common pathogens and cumulative infection burden with neurocognitive disorder (mild cognitive impairment and dementia)
Shiota 2011	Japan	Case-control	917	AD	using a rapid urine test (RAPIRUN H. pylori antibody)	n/a	Prevalence of H. pylori infection in patients with AD and controls
Tsolaki 2015	Greece	Case-control	156	AD	Gastroscopy, histological examination of the retrieved tissue specimens and serological examination of IgG antibodies against H.pylori	n/a	Association between Alzheimer’s disease and other dementias (frontotemporal- dementia- FTD), dementia with Parkinson’s disease-PD, Lewy body dementia-LB), primary open-angle glaucoma (POAG) and H.pylori infection in all possible combinations,

*n/a* Not Available, *AD* Alzheimer’s Disease, *CD* Cognitive decline, *US* United States, *UK* United Kingdom

**Table 2:** Baseline of the included studies

Study ID	Study groups, (*n*)	Age, Mean ± SD	Sex (male), NO. (%)	BMI, Mean ± SD	Medical history						Smoking		Education	
					Hypertension, NO. (%)		Atrial fibrillation, NO. (%)	Coronary artery disease, NO.(%)	Cerebrovascular disease, NO. (%)	Diabetes, NO. (%)	Yes, NO. (%)	Cigarettes/day, Mean ± SD	Education (year), Mean ± SD	More Than High School, NO. (%)
Beydoun 2018	H.pylori Negative (2707)	59.3 ± 10.4	1250 (46.2)	n/a	1305 (48.2)		n/a	n/a	n/a	300 (11.1)	541 (20)	n/a	n/a	1296 (47.9)
	H.pylori Positive (3220)	62.8 ± 22.7	1458 (45.3)	n/a	1916 (59.5)		n/a	n/a	n/a	502 (15.6)	760 (23.6)	n/a	n/a	814 (25.3)
Beydoun 2020	H.pylori Negative (558)	72.70 ± 9.2	222 (39.77)	n/a	High SBP	271 (48.63)	n/a	n/a	n/a	66 (11.87)	n/a	4.63 ± 15.1	11.86 ± 5.9	n/a
					High DBP	29 (5.24)								
	H.pylori Positive (881)	73.76 ± 9.5	364 (41.35)	n/a	High SBP	445 (50.49)	n/a	n/a	n/a	135 (15.37)	n/a	5.99 ± 15.7	10.47 ± 7.1	n/a
					High DBP	47 (5.33)								
Bu 2015	Controls (135)	69 ± 9	72 (53.3)	n/a	46 (34.1)		n/a	19 (14.1)	n/a	21 (15.6)	n/a	n/a	n/a	102 (75.6)
	AD (128)	70 ± 10	59 (46.1)	n/a	48 (37.5)		n/a	24 (18.8)	n/a	26 (20.3)	n/a	n/a	n/a	81 (63.3)
Cardenas 2019	Overall (2892)	69.83 ± 9	1474 (51)	n/a	1811 (62.2)		n/a	n/a	n/a	433 (14.97)	n/a	n/a	n/a	712 (24.6)
Douros 2024	AD (40,455)	69 ± 8.8	14,454 (35.7)	n/a	11,814 (29.2)		964 (2.4)	4509 (11.1)	1242 (3.1)	2440 (6.0)	12,855 (31.8)	n/a	n/a	n/a
	Controls (1,610,502)	69 ± 8.8	573,799 (35.6)	n/a	492,501 (30.6)		43,692 (2.7)	173,760 (10.8)	59,904 (3.7)	93,420 (5.8)	490,181 (30.4)	n/a	n/a	n/a
Fu 2024	Dementia (107)	65.3 ± 4.5	57 (53.27)	26.54 ± 2.94	Cardiovascular disease 56 (52.34)				n/a	16 (14.95)	n/a	n/a	n/a	61 (57.01)
	Non-dementia (8,037)	57 ± 9.64	3506 (43.62)	26.76 ± 4.23	2,236 (27.82)				n/a	369 (4.59)	n/a	n/a	n/a	3983 (49.56)
	AD (55)	64.67 ± 5.33	18 (32.73)	26.68 ± 3.52	26 (47.27)				n/a	9 (16.36)	n/a	n/a	n/a	36 (65.45)
	Non-AD (8,089)	57 ± 9.64	3545 (43.82)	26.75 ± 4.21	2,266 (28.01)				n/a	376 (4.65)	n/a	n/a	n/a	6764 (83.62)
Han 2017	Q1 (147)	72.6 ± 5.0	73 (49.67)	23.7 ± 2.7	95 (64.6)		n/a	n/a	n/a	25 (17)	25 (17)	n/a	13.2 ± 3.9	n/a
	Q2 (146)	73.8 ± 5.9	78 (53.42)	24.1 ± 3.2	105 (71.9)		n/a	n/a	n/a	26 (17.8)	23 (15.75)	n/a	13.4 ± 4.2	n/a
	Q3 (148)	72.9 ± 5.9	82 (55.4)	24.0 ± 3.3	98 (66.2)		n/a	n/a	n/a	29 (19.6)	29 (19.6)	n/a	13.4 ± 3.6	n/a
	Q4 (146)	73.0 ± 5.6	79 (54.11)	24.1 ± 2.7	107 (73.29)		n/a	n/a	n/a	17 (11.64)	19 (13.01)	n/a	13.5 ± 4.1	n/a
Huang 2014	H. Pylori Negative (67,172)	63.2 ± 13.5	41368 (61.6)	n/a	11,056 (16.5)		n/a	n/a	n/a	5824 (8.67)	n/a	n/a	n/a	n/a
	H. Pylori Positive (16,793)	63.6 ± 13.4	10342 (61.6)	n/a	4925 (29.3)		n/a	n/a	n/a	2914 (17.4)	n/a	n/a	n/a	n/a
Kountouras 2006	AD (50)	65.0 ± 6.9	18 (36)	n/a	n/a		n/a	n/a	n/a	n/a	n/a	n/a	n/a	n/a
	Anemic controls (30)	62.2 ± 8.6	14 (46.67)	n/a	n/a		n/a	n/a	n/a	n/a	n/a	n/a	n/a	n/a
Koyama 2016	Cognitive decline (34)	61.2 ± 4.6	19 (55.9)	22.6 ± 3.3	14 (41.2)		n/a	n/a	n/a	8 (23.5)	16 (47)	n/a	n/a	11 (32.4)
	Control (117)	59.4 ± 5.9	82 (70.1)	22.3 ± 2.4	35 (29.9)		n/a	n/a	n/a	21 (18)	61 (52.1)	n/a	n/a	60 (51.3)
Lu 2022	Overall (884)	67.9 ± 8.1	361 (40.8)	n/a	n/a		n/a	n/a	n/a	n/a	190 (21.5)	n/a	n/a	333 (37.7)
Rezaeimehr 2016	H. Pylori Positive (758)	69.3 ± 7.4	594 (78.3)	n/a	606 (75.0)		n/a	n/a	n/a	303 (77.5)	n/a	n/a	n/a	?
	H. Pylori Negative (756)		165 (21.7)	n/a	202 (25.0)		n/a	n/a	n/a	88 (22.5)	n/a	n/a	n/a	?
Roubaud-Baudron 2012	Patients (20)	70.1 ± 8.1	6 (30)	25.2 5.4	9(45)		n/a	n/a	n/a	n/a	3(15)	n/a	n/a	n/a
	Controls (33)	66.9 ± 9.0	17 (52)	24.2 4.4	12(36)		n/a	n/a	n/a	n/a	8(24)	n/a	n/a	n/a
Shi 2024	Overall (475)	67.57 ± 7.94	196 (41.3)	n/a	n/a		n/a	n/a	n/a	n/a	n/a	n/a	n/a	223 (46.9)
Shiota 2011	AD (385)	78.5 ± 6.4	114 (29.6)	n/a	n/a		n/a	n/a	n/a	n/a	n/a	n/a	n/a	n/a
	Controls (97)	70.4 ± 9.8	17 (17.5)	n/a	n/a		n/a	n/a	n/a	n/a	n/a	n/a	n/a	n/a
Tsolaki 2015	Patients with dementia (60)	61.34 ± 6.526	28 (46.66)	n/a	n/a		n/a	n/a	n/a	n/a	n/a	n/a	n/a	n/a
	Patients with glaucoma (35)	62.18 ± 5.04	14 (46.66)	n/a	n/a		n/a	n/a	n/a	n/a	n/a	n/a	n/a	n/a
	First control (31)	62.41 ± 4.49	14 (45.16)	n/a	n/a		n/a	n/a	n/a	n/a	n/a	n/a	n/a	n/a
	Second control (30)	61.48 ± 2.8	13 (43.44)	n/a	n/a		n/a	n/a	n/a	n/a	n/a	n/a	n/a	n/a

*n/a* Not available, *NO*. Number, *SD* Standard Deviation

### Quality assessment

The assessment indicated that all cohort studies were rated as"Good,"while two case-control and one cross-sectional study received a"Fair"rating due to Limitations in variable domains. Details of the quality assessment are demonstrated in Supplementary Tables 2–4.

### Meta-analysis

#### H. pylori-positive cohorts and risk of cognitive decline

**Fig. 2:**
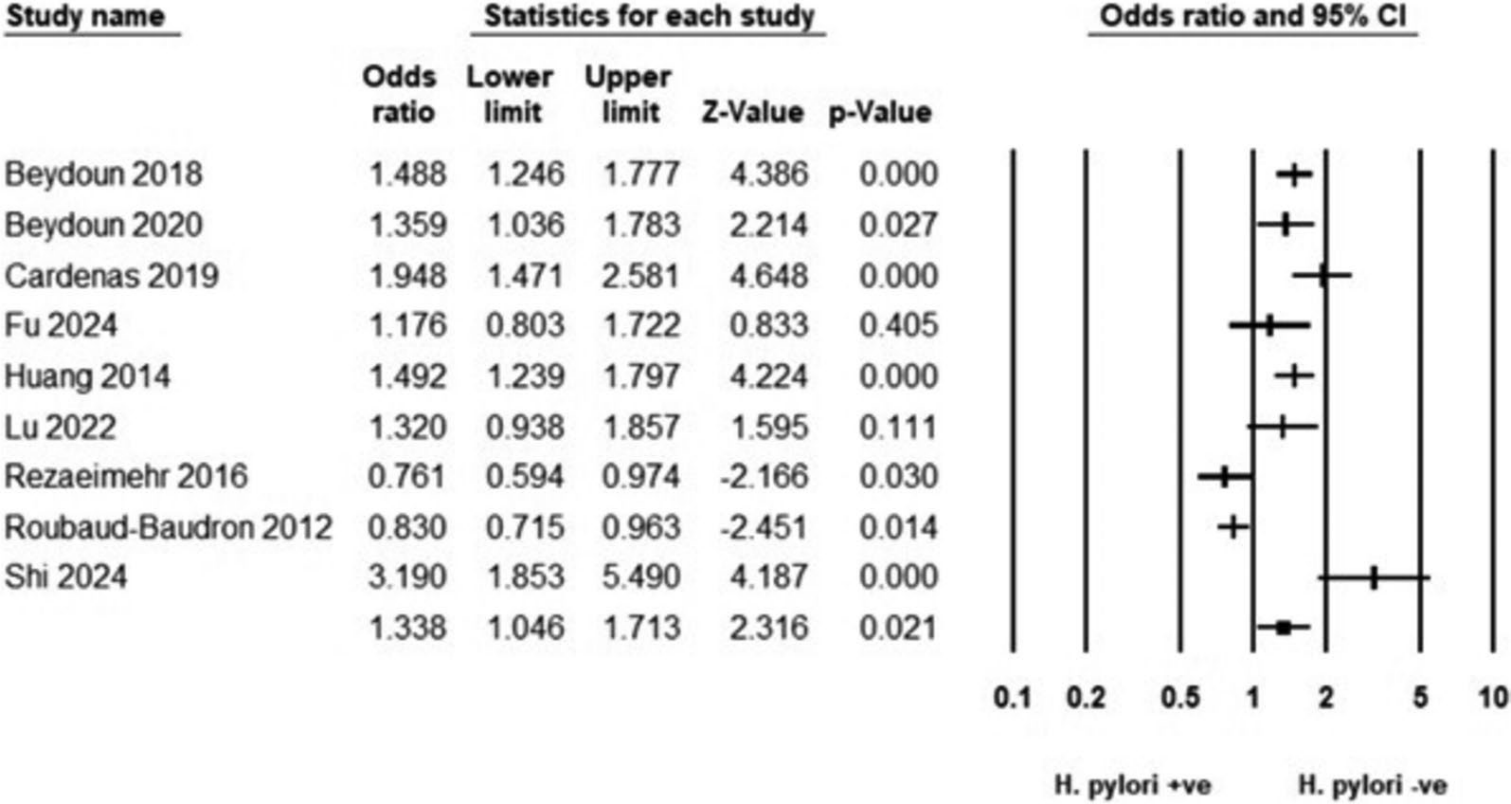
Forest plot illustrating the pooled association between H. pylori infection and cognitive decline risk

**Fig. 3:**
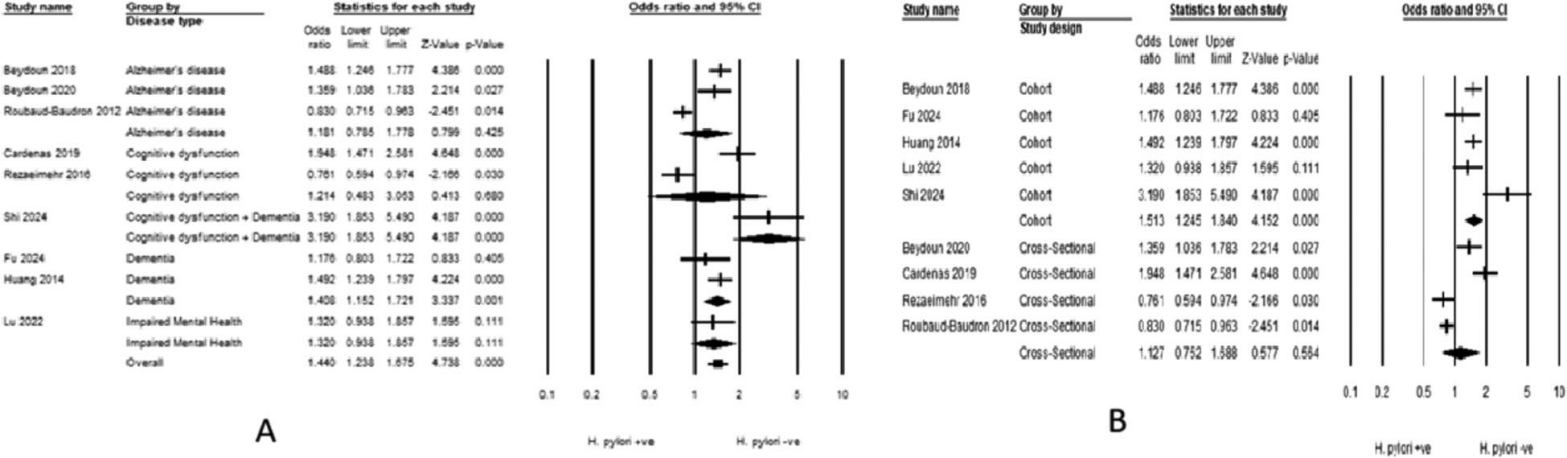
Forest plot illustrating the pooled association between H. pylori infection and cognitive decline risk, stratified by disease definitions and by study designs

Patients with H. pylori infection had a significantly higher prevalence of cognitive decline compared to those without the infection (OR = 1.338, 95% CI 1.046–1.713) (Fig. 2). Heterogeneity was considerable (*I*^2^ = 88.95%). The subgroup analysis based on disease definition showed varying associations. The strongest association was observed in the study that grouped cognitive dysfunction and dementia (OR: 3.190, 95% CI 1.853–5.490), followed by dementia alone (OR: 1.408, 95% CI 1.152–1.721). In contrast, AD showed no significant association (OR: 1.181, 95% CI 0.785–1.778). Similarly, cognitive dysfunction and impaired mental health showed no significant association (OR: 1.214, 95% CI 0.483–3.053) and (OR: 1.320, 95% CI 0.938–1.857), respectively. Considerable heterogeneity persisted in AD studies (*I*^2^ = 92.64%) and cognitive dysfunction studies (*I*^2^ = 95.97%) (Fig. 3A).

The subgroup analysis based on study design revealed that cohort studies consistently showed a significant association between H. pylori infection and cognitive decline risk, with a pooled odds ratio of 1.513 (95% CI 1.245–1.840). In contrast, cross-sectional studies showed no significant association, with ORs of 1.127 (95% CI 0.752–1.688). Heterogeneity was substantial in cohort studies (*I*^2^ = 57.95%) and considerable in cross-sectional studies (*I*^2^ = 91.94%) (Fig. 3B).

**Fig. 4:**
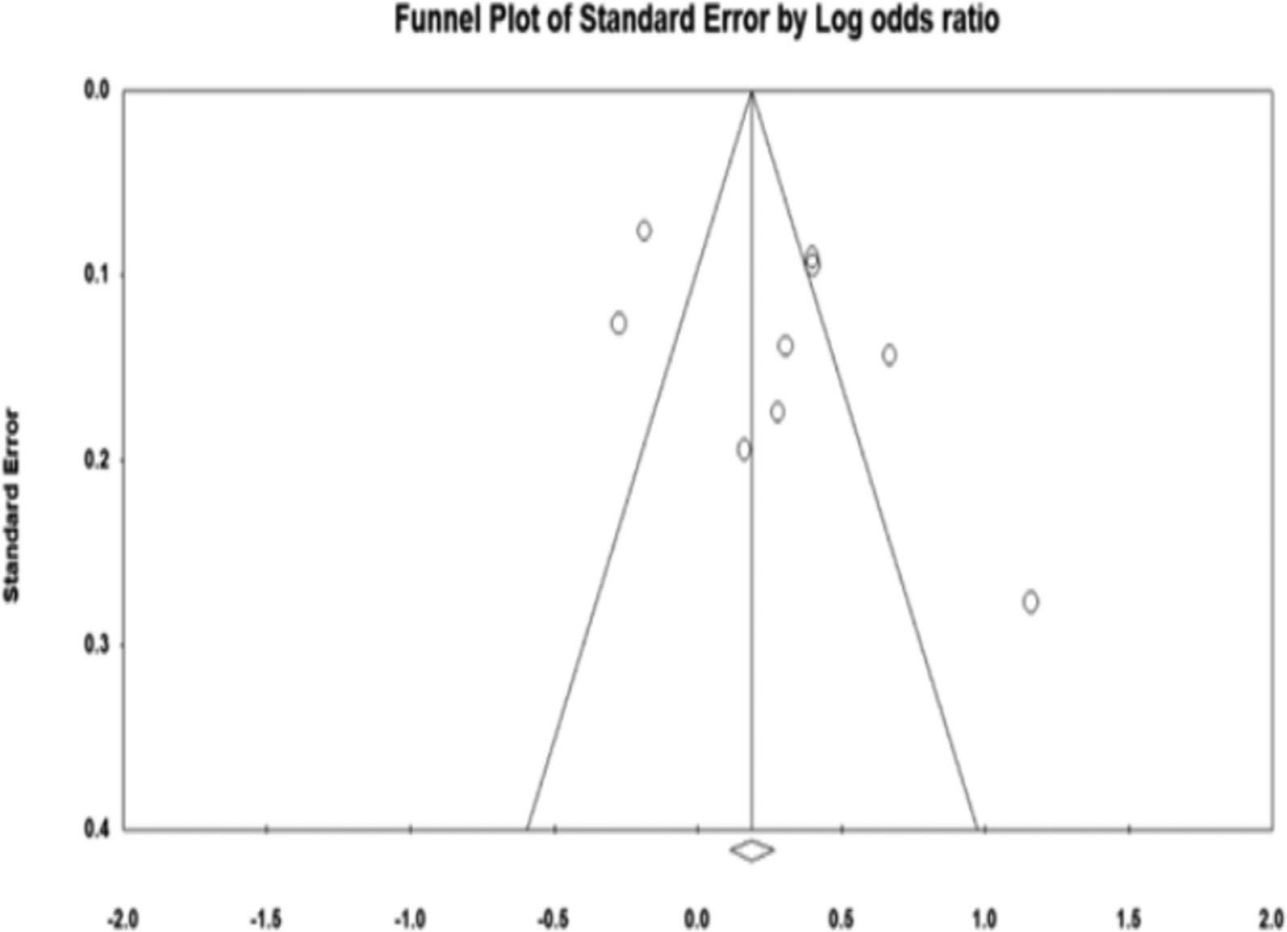
Funnel plot assessing publication bias in the meta-analysis of cognitive decline risk in patients with H. pylori infection

The funnel plot was symmetrical, and Egger’s regression test (p = 0.21) confirmed no evidence of publication bias (Fig. 4).

#### Cognitive decline cohorts and prevalence of H. pylori infection

**Fig. 5:**
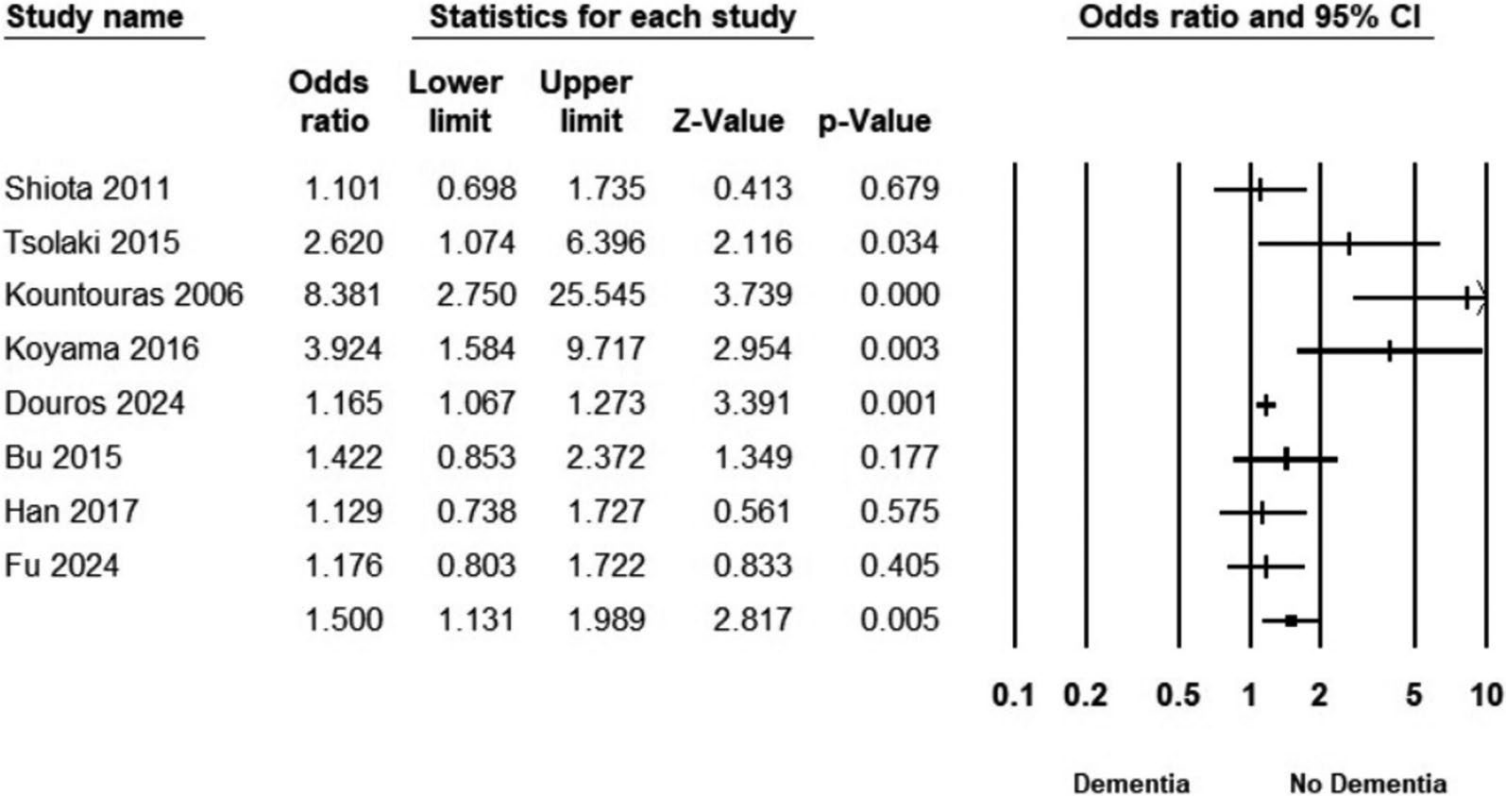
Forest plot illustrating the pooled association between risk of H. pylori infection and cognitive decline

**Fig. 6:**
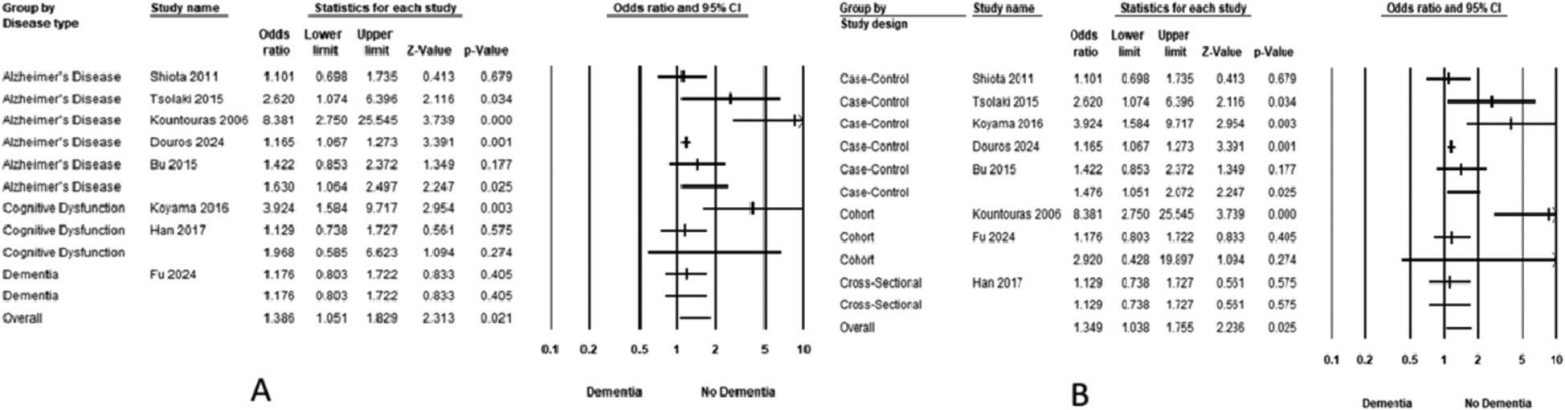
Forest plot illustrating the pooled association between risk of H. pylori infection and cognitive decline, stratified by disease definitions and by study designs

Patients with cognitive decline had a significantly higher prevalence of H. pylori infection than those without cognitive decline (OR = 1.5, 95% CI 1.131–1.989) (Fig. 5). Heterogeneity was substantial (*I*^2^ = 68.55%). The subgroup analysis revealed varying associations between H. pylori infection and different types of cognitive impairment. The only significant association was observed in the AD subgroup (OR: 1.630, 95% CI 1.064–2.497). No significant association was observed in cognitive dysfunction subgroups or dementia: (OR: 1.968, 95% CI 0.585–6.623) and (OR: 1.176, 95% CI 0.803–1.722). Heterogeneity remained considerable even after subgrouping, with AD studies showing *I*^2^ = 74.3% and cognitive dysfunction studies exhibiting *I*^2^ = 83.2% (Fig. 6A).

The stratification of the studies based on the study design yielded a significant association with case-control studies (OR: 1.476, 95% CI 1.051–2.072). In contrast, cohort studies (OR: 2.920, 95% CI 0.428–19.897) and cross-sectional studies (OR: 1.129, 95% CI 0.738–1.727) did not show statistically significant associations (Fig. 6B). Similarly, heterogeneity was not resolved.

**Fig. 7:**
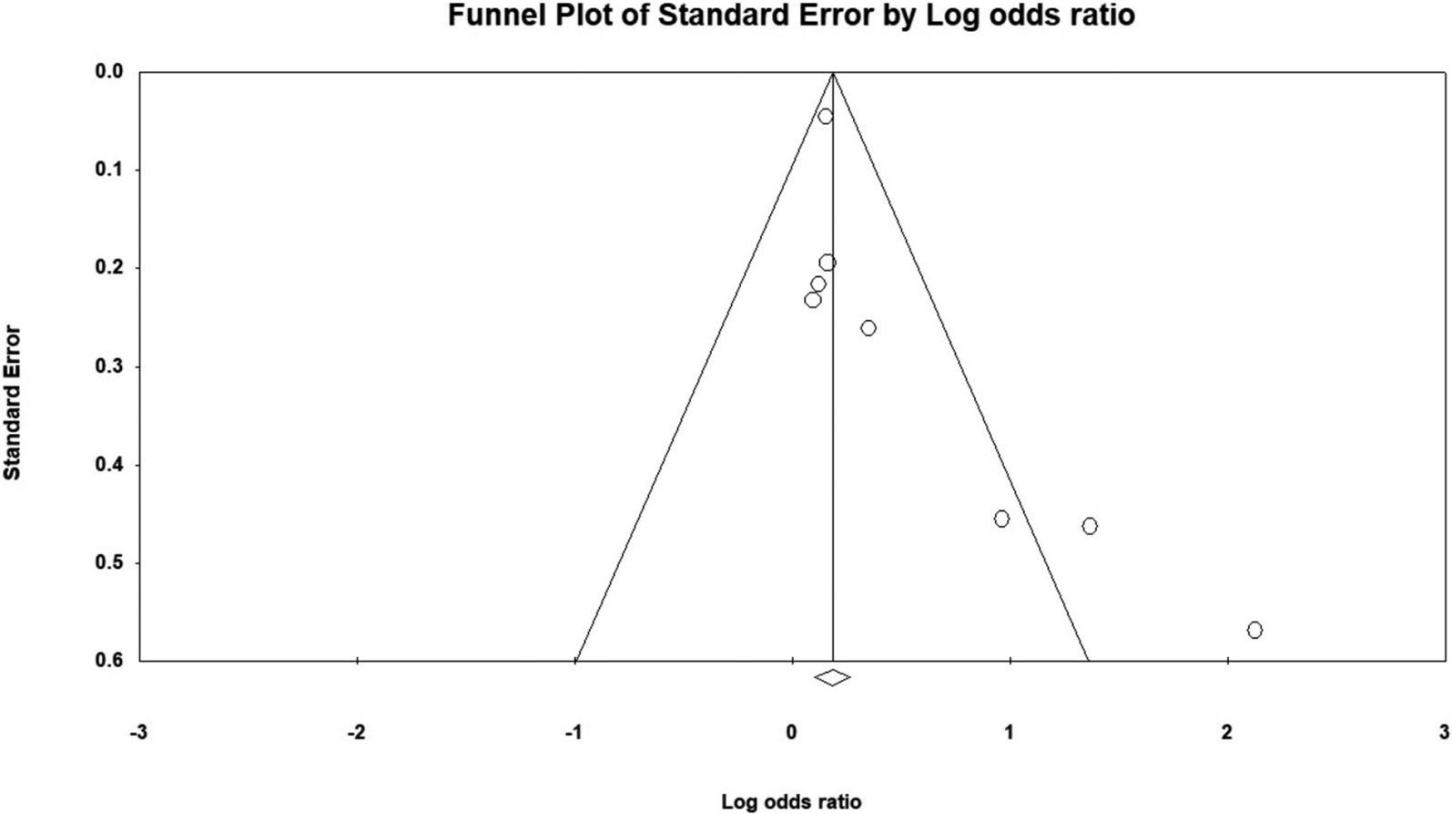
Funnel plot assessing publication bias in the meta-analysis of H. pylori infection risk in patients with cognitive decline

There is a possibility of borderline publication bias, as suggested by the asymmetrical funnel plot and Egger's regression test (*p* = 0.055) (Fig. 7).

#### Meta-regression analysis

Meta-regression analysis was conducted to explore potential moderators influencing the association between H. pylori infection and cognitive impairment (Table 3). For studies examining the prevalence of H. pylori among cognitively impaired cohorts, age (categorized as < 70 vs. ≥ 70 years based on baseline mean age) did not show a statistically significant moderating effect (*p* = 0.1467), though it explained 20% of the variance, with substantial heterogeneity observed (*I*^2^ = 85.58%). Country also did not significantly moderate the effect (p > 0.05), But it accounted for 90% of the variance with lower heterogeneity (*I*^2^ = 45.1%). In contrast, for studies assessing the risk of cognitive decline in H. pylori-positive cohorts, age showed a statistically significant moderating effect (*p* = 0.027), though it explained only 6% of the variance and had moderate heterogeneity (*I*^2^ = 63.07%). Country did not significantly moderate this association (*p* = 0.086) and explained none of the variance (*R*^2^ = 0.00), with an *I*^2^ of 68.55%.

**Table 3:** Meta-regression analysis of moderators influencing the association between H. pylori and cognitive impairment

Outcome	Covariate/Moderator	*P*-value (significant moderating effect)	Between-study heterogeneity (I^2^)	Explained variance (*R*^2^ analog)
Association (H-pylori status)	Age**	0.1467	85.58%	0.2
	Country	> 0.05	45.1%	0.9
Association (Dementia status)	Age**	0.027	63.07%	0.06
	Country	0.086	68.55%	0.00

^**^Age is categorized to (Less than 70) OR (70 or more) according to age mean in baseline

## Discussion

In this meta-analysis, we explored the bidirectional relationship between H. pylori infection and cognitive decline. The pooled results suggested a significant overall association in both directions, though the strength and consistency of this relationship varied across diagnostic definitions and study designs. Notably, a stronger and more consistent link was observed in longitudinal cohorts assessing dementia risk in infected individuals, particularly for non-Alzheimer’s forms of cognitive impairment. In contrast, studies evaluating H. pylori prevalence among dementia patients yielded more mixed findings, with some evidence of association in AD subgroups. However, persistent heterogeneity and methodological differences across included studies limit definitive conclusions.

Our findings align with a previous meta-analysis by Liu et al., which included 10 studies and reported a significant association between H. pylori infection and all-cause dementia, but not AD [27]. Our study Builds upon and extends their work by including 16 studies with more recent data and adopting a bidirectional approach—assessing both the risk of cognitive decline in infected individuals and the prevalence of H. pylori in cognitively impaired patients. This dual analysis offers a more comprehensive assessment of the association. Additionally, we performed detailed subgroup analyses based on study design and diagnostic categories, which helped identify patterns and sources of heterogeneity, providing more nuanced insights into the relationship.

Several biological mechanisms have been suggested to explain the link between H. pylori infection and cognitive decline. Chronic inflammation has been suggested as the primary pathway for this association. Inflammatory neurotoxic cytokines are released during H. pylori infection, leading to neuronal apoptosis and consequent neurodegeneration [50, 51]. Structural components of H. pylori itself also exhibit pro-inflammatory properties. For example, H. pylori [2–20] peptides interact with amyloid-β42 and formyl peptide receptors, activating inflammatory pathways that promote neurodegeneration. This interaction has been linked to an increase in vascular endothelial growth factor (VEGF), a factor associated with both vascular dementia and neurodegenerative diseases [52]. Additionally, H. pylori was observed to significantly induce tau hyperphosphorylation at multiple AD-related phosphorylation sites [53].

Beyond inflammation, H. pylori infection affects metabolic pathways associated with neurodegeneration. It has been associated with metabolic syndrome, characterized by elevated homocysteine levels and reduced vitamin B12 and 5-methyltetrahydrofolate, leading to endothelial damage and vascular dysfunction, both key contributors to cognitive decline [54, 55]. Notably, lower 5-methyltetrahydrofolate levels in H. pylori-infected individuals further reinforce the role of metabolic dysregulation in cognitive impairment [56]. These findings suggest that H. pylori eradication may have therapeutic benefits in neurodegenerative diseases, a hypothesis warranting further investigation.

A previous study, by Chang and colleagues, reported that H. pylori eradication was linked to slower dementia progression in AD patients with peptic ulcers compared to those who did not receive eradication treatment [57]. Interestingly, a recent study in Korea found that in patients with peptic ulcer disease (PUD) and H. pylori infection, delayed eradication was associated with a significantly higher risk of developing dementia, including AD, compared to early eradication [58]. However, these conclusions remain preliminary. Previous studies have shown that H. pylori eradication is linked to improvements in cognitive and functional status, as well as increased survival rates in patients with AD [59, 60]. From a clinical perspective, the association between H. pylori infection and cognitive decline presents two key considerations for healthcare providers. First, eradicating H. pylori, even in asymptomatic individuals, may help reduce the risk of cognitive decline, though further high-quality research is needed to confirm this potential benefit. Second, recognizing this link allows clinicians to investigate whether screening for H. pylori and its eradication influence the progression of existing cognitive decline, shaping future preventive and therapeutic strategies.

Conflicting findings challenge the temporal direction of this association. A recent large-scale, nested case-control study conducted in Finland examined this relationship in over 70,000 individuals diagnosed with AD and age-, sex-, and region-matched controls [61]. Eradication of H. pylori showed no significant association with decreased AD, with an odds ratio close to one after some potential confounders were considered. This evidence indicates that H. pylori infection is not an independent risk factor for AD. One possible explanation for these discrepancies is reverse causation, where early cognitive impairment may increase susceptibility to infections rather than H. pylori contributing to dementia onset. Additionally, confounding factors, such as lifestyle, socioeconomic status, and underlying health conditions, may influence both H. pylori infection and cognitive decline, complicating causal inference. However, this study only focused on AD without consideration of other stages of cognitive decline and other dementia types. Our meta-analysis found that patients with H. pylori infection do not have a higher risk of developing AD compared to those without the infection. However, among patients with AD, the prevalence of H. pylori was significantly higher. This finding may provide insights into the possibility of reverse causation between AD and H. pylori, as suggested by the previous study. Further research is needed to clarify the nature of the relationship between H. pylori infection and cognitive decline. Longitudinal studies with better control of confounding factors are essential to determine whether H. pylori plays a causal role in dementia. Additionally, mechanistic studies should explore whether pathways such as neuroinflammation and hyperhomocysteinemia mediate this association. Finally, randomized controlled trials investigating whether H. pylori eradication influences cognitive outcomes would provide valuable evidence on its potential role in dementia prevention.

This meta-analysis comprehensively pooled the available evidence on the bidirectional association between H. pylori infection and cognitive decline. Through subgroup analyses, we identified areas where the association was significant and others where it was not, providing insights for future research. While the findings suggest that H. pylori may contribute to cognitive decline in a broader sense, encompassing various forms of dementia, the evidence does not support H. pylori as an independent risk factor for AD. The observed differences across study designs further highlight the complexity of this relationship, which may be influenced by methodological variations and confounding factors. There are various limitations to be acknowledged. This meta-analysis employs only observational studies; therefore, it cannot draw causal conclusions about the relationship between H. pylori infection and the risk of cognitive decline, and thus requires careful interpretation of its results. Despite subgroup analyses, considerable heterogeneity remained across studies. Meta-regression was conducted to explore potential sources of this heterogeneity. In the analysis of cognitive decline prevalence and H. pylori infection, age (categorized as < 70 vs. ≥ 70 years) was not a statistically significant moderator (*p* = 0.1467), although it explained 20% of the variance. Similarly, the country of origin showed a non-significant moderating effect (*p* > 0.05), but it accounted for a larger portion of heterogeneity (*R*^2^ analog = 0.9), suggesting geographic or population-level factors may still influence results. In contrast, among H. pylori-positive cohorts, age was a significant moderator of the risk of cognitive decline (*p* = 0.027), though it explained only 6% of the variance. Country showed a borderline effect (*p* = 0.086) but did not explain heterogeneity (*R*^2^ analog = 0.00).

These findings highlight that age may be a relevant effect modifier in determining the cognitive risk among infected individuals. Nonetheless, residual heterogeneity persisted (*I*^2^ ranging from 45 to 85%), likely due to variations in study populations, diagnostic criteria for cognitive decline, H. pylori detection methods, and inconsistent adjustment for confounders. Cognitive decline is multifactorial, influenced by comorbidities (e.g., cardiovascular disease, diabetes), socioeconomic status, and healthcare access—all of which may also affect H. pylori prevalence and its detection. Additionally, prior eradication therapy, rarely reported, could modify infection status and its cognitive impact. Future studies should adjust for these factors, stratify analyses by eradication status, and consider individual-level data to reduce confounding and clarify the nature of this association.

Finally, in studies evaluating the prevalence of H. pylori among patients with cognitive impairment, signs of borderline publication bias were noted. This suggests a possible small-study effect or selective reporting of positive results, which could lead to an overestimation of the true association. Therefore, findings from these analyses should be interpreted with appropriate caution.

## Conclusion

This meta-analysis suggests a potential bidirectional association between H. pylori infection and cognitive decline. However, the strength and consistency of this relationship varied depending on the definitions of cognitive impairment and study design. While several studies indicated an increased risk of cognitive decline—particularly non-Alzheimer’s dementia—in individuals with H. pylori infection, no consistent association was found with Alzheimer’s disease. Conversely, a higher prevalence of H. pylori infection among patients with Alzheimer’s disease may reflect reverse causation rather than a causal relationship. Substantial heterogeneity and the observational nature of the included studies limit the certainty of these findings. Therefore, conclusions should be interpreted with caution. Future research should prioritize well-controlled, multi-center longitudinal cohort studies and randomized controlled trials—where feasible—to establish temporality and causality. Additionally, studies should standardize diagnostic criteria, adjust for key confounders (e.g., age, comorbidities, socioeconomic status), and account for prior eradication therapy. Subtype-specific analyses of dementia are also warranted to better understand underlying mechanisms.

## Supplementary Information

Supplementary material 1.

## Data Availability

No datasets were generated or analysed during the current study.

## References

[1] Gale SA, Acar D, Daffner KR. Dementia. Am J Med. 2018;131(10):1161–9. 29425707 10.1016/j.amjmed.2018.01.022

[2] Knopman DS, Petersen RC. Mild cognitive impairment and mild dementia: a clinical perspective. Mayo Clin Proc. 2014;89(10):1452–9. 25282431 10.1016/j.mayocp.2014.06.019 PMC4185370

[3] Nichols E, Steinmetz JD, Vollset SE, Fukutaki K, Chalek J, Abd-Allah F, et al. Estimation of the global prevalence of dementia in 2019 and forecasted prevalence in 2050: an analysis for the Global Burden of Disease Study 2019. Lancet Public Health. 2022;7(2):e105–25. 34998485 10.1016/S2468-2667(21)00249-8 PMC8810394

[4] 2024 Alzheimer’s disease facts and figures. Alzheimers Dement. 2024;20(5):3708–821. 10.1002/alz.13809. 10.1002/alz.13809 PMC11095490 38689398

[5] Arvanitakis Z, Shah RC, Bennett DA. Diagnosis and management of dementia: review. JAMA. 2019;322(16):1589. 31638686 10.1001/jama.2019.4782 PMC7462122

[6] Lastuka A, Bliss E, Breshock MR, Iannucci VC, Sogge W, Taylor KV, et al. Societal costs of Dementia: 204 Countries, 2000–2019. J Alzheimer’s Dis. 2024;101(1):277–92. 39150827 10.3233/JAD-240163 PMC11380273

[7] Dintica CS, Yaffe K. Epidemiology and risk factors for dementia. Psychiatr Clin North Am. 2022;45(4):677–89. 36396272 10.1016/j.psc.2022.07.011

[8] Van Nieuwkerk AC, Delewi R, Wolters FJ, Muller M, Daemen M, Biessels GJ, et al. Cognitive impairment in patients with cardiac disease: implications for clinical practice. Stroke. 2023;54(8):2181–91. 37272393 10.1161/STROKEAHA.123.040499

[9] Cao F, Yang F, Li J, Guo W, Zhang C, Gao F, et al. The relationship between diabetes and the dementia risk: a meta-analysis. Diabetol Metab Syndr. 2024;16(1):101. 38745237 10.1186/s13098-024-01346-4 PMC11092065

[10] Kuźma E, Lourida I, Moore SF, Levine DA, Ukoumunne OC, Llewellyn DJ. Stroke and dementia risk: a systematic review and meta-analysis. Alzheimers Dement. 2018;14(11):1416–26. 30177276 10.1016/j.jalz.2018.06.3061 PMC6231970

[11] Zhao M, Ma G, Yan X, Li X, Wang E, Xu XX, et al. Microbial infection promotes amyloid pathology in a mouse model of Alzheimer’s disease via modulating γ-secretase. Mol Psychiatry. 2024;29(5):1491–500. 38273109 10.1038/s41380-024-02428-5

[12] Li Z, Wang H, Yin Y. Peripheral inflammation is a potential etiological factor in Alzheimer’s disease. Rev Neurosci. 2024;35(1):99–120. 37602685 10.1515/revneuro-2023-0049

[13] Olivera E, Sáez A, Carniglia L, Caruso C, Lasaga M, Durand D. Alzheimer’s disease risk after COVID-19: a view from the perspective of the infectious hypothesis of neurodegeneration. Neural Regen Res. 2023;18(7):1404. 36571334 10.4103/1673-5374.360273 PMC10075115

[14] Bruno F, Abondio P, Bruno R, Ceraudo L, Paparazzo E, Citrigno L, et al. Alzheimer’s disease as a viral disease: revisiting the infectious hypothesis. Ageing Res Rev. 2023;91:102068. 37704050 10.1016/j.arr.2023.102068

[15] Mody PH, Marvin KN, Hynds DL, Hanson LK. Cytomegalovirus infection induces Alzheimer’s disease-associated alterations in tau. J Neurovirol. 2023;29(4):400–15. 37436577 10.1007/s13365-022-01109-9

[16] Dominy SS, Lynch C, Ermini F, Benedyk M, Marczyk A, Konradi A, et al. *Porphyromonas**gingivalis* in Alzheimer’s disease brains: evidence for disease causation and treatment with small-molecule inhibitors. Sci Adv. 2019;5(1):eaau3333. 30746447 10.1126/sciadv.aau3333 PMC6357742

[17] Honjo K, Van Reekum R, Verhoeff NPLG. Alzheimer’s disease and infection: do infectious agents contribute to progression of Alzheimer’s disease? Alzheimers Dement. 2009;5(4):348–60. 19560105 10.1016/j.jalz.2008.12.001

[18] Liccardo D, Marzano F, Carraturo F, Guida M, Femminella GD, Bencivenga L, et al. Potential bidirectional relationship between periodontitis and Alzheimer’s disease. Front Physiol. 2020;11:683. 32719612 10.3389/fphys.2020.00683 PMC7348667

[19] Chen YC, Malfertheiner P, Yu HT, Kuo CL, Chang YY, Meng FT, et al. Global prevalence of *Helicobacter pylori* infection and incidence of gastric cancer between 1980 and 2022. Gastroenterology. 2024;166(4):605–19. 38176660 10.1053/j.gastro.2023.12.022

[20] Hooi JKY, Lai WY, Ng WK, Suen MMY, Underwood FE, Tanyingoh D, et al. Global prevalence of *Helicobacter pylori* infection: systematic review and meta-analysis. Gastroenterology. 2017;153(2):420–9. 28456631 10.1053/j.gastro.2017.04.022

[21] Alzahrani S. Effect of *Helicobacter pylori* on gastric epithelial cells. World J Gastroenterol. 2014;20(36):12767. 25278677 10.3748/wjg.v20.i36.12767 PMC4177462

[22] Hu Q, Zhang Y, Zhang X, Fu K. Gastric mucosa-associated lymphoid tissue lymphoma and Helicobacter pylori infection: a review of current diagnosis and management. Biomark Res. 2016;4(1):15. 27468353 10.1186/s40364-016-0068-1 PMC4962427

[23] McConaghy JR, Decker A, Nair S. Peptic ulcer disease and H. pylori infection: common questions and answers. Am Fam Physician. 2023;107(2):165–72. 36791443

[24] Rezaeimehr Z, Hosseini SR, Darbandi Z, Hosseini SA, Kheirkhah F, Bijani A, et al. The association between helicobacter pylori infection and cognitive disorder in Iranian elderly population. Arch Clin Infect Dis. 2016;11(4):e38193.

[25] Douros A, Ante Z, Fallone CA, Azoulay L, Renoux C, Suissa S, et al. Clinically apparent *Helicobacter pylori* infection and the risk of incident Alzheimer’s disease: a population-based nested case-control study. Alzheimers Dement. 2024;20(3):1716–24. 38088512 10.1002/alz.13561 PMC10984501

[26] Fu J, Wei Q, Chen X, Lai X, Shang H. Analysis of the association between pathogen exposure and the risk of dementia. J Alzheimers Dis. 2024;100(3):961–72. 38995782 10.3233/JAD-240073

[27] Liu NY, Sun JH, Jiang XF, Li H. *Helicobacter pylori* infection and risk for developing dementia: an evidence-based meta-analysis of case-control and cohort studies. Aging. 2021;13(18):22571–87. 34559067 10.18632/aging.203571 PMC8507304

[28] Xie J, Van Hoecke L, Vandenbroucke RE. The impact of systemic inflammation on Alzheimer’s disease pathology. Front Immunol. 2022;12:796867. 35069578 10.3389/fimmu.2021.796867 PMC8770958

[29] Baj J, Forma A, Flieger W, Morawska I, Michalski A, Buszewicz G, et al. *Helicobacter pylori* infection and extragastric diseases—a focus on the central nervous system. Cells. 2021;10(9):2191. 34571840 10.3390/cells10092191 PMC8469861

[30] Kountouras J, Doulberis M, Papaefthymiou A, Polyzos SA, Zavos C, Kazakos E, et al. Controlling the impact of *Helicobacter pylori*-related hyperhomocysteinemia on neurodegeneration. Medicina (Mex). 2023;59(3):504. 10.3390/medicina59030504 PMC10056452 36984505

[31] Page MJ, McKenzie JE, Bossuyt PM, Boutron I, Hoffmann TC, Mulrow CD, et al. The PRISMA 2020 statement: an updated guideline for reporting systematic reviews. BMJ. 2021;29:n71. 10.1136/bmj.n71 PMC8005924 33782057

[32] Ottawa Hospital Research Institute. https://www.ohri.ca/programs/clinical_epidemiology/oxford.asp. Accessed 25 Feb 2025.

[33] Ribeiro CM, Beserra BTS, Silva NG, Lima CL, Rocha PRS, Coelho MS, et al. Exposure to endocrine-disrupting chemicals and anthropometric measures of obesity: a systematic review and meta-analysis. BMJ Open. 2020;10(6):e033509. 32565448 10.1136/bmjopen-2019-033509 PMC7311014

[34] Higgins JPT. Measuring inconsistency in meta-analyses. BMJ. 2003;327(7414):557–60. 12958120 10.1136/bmj.327.7414.557 PMC192859

[35] Quinn BP. Diagnostic and statistical manual of mental disorders, fourth edition, primary care version. Prim Care Companion J Clin Psychiatry. 1999;1(2):54–5.

[36] Egger M, Smith GD, Schneider M, Minder C. Bias in meta-analysis detected by a simple, graphical test. BMJ. 1997;315(7109):629–34. 9310563 10.1136/bmj.315.7109.629 PMC2127453

[37] Tsolaki F, Kountouras J, Topouzis F, Tsolaki M. *Helicobacter**pylori* infection, dementia and primary open-angle glaucoma: are they connected? BMC Ophthalmol. 2015;15(1):24. 25880776 10.1186/s12886-015-0006-2 PMC4379965

[38] Shiota S, Murakami K, Yoshiiwa A, Yamamoto K, Ohno S, Kuroda A, et al. The relationship between *Helicobacter pylori* infection and Alzheimer’s disease in Japan. J Neurol. 2011;258(8):1460–3. 21336779 10.1007/s00415-011-5957-5 PMC3742106

[39] Shi R, Yu S, Larbi A, Pin Ng T, Lu Y. Specific and cumulative infection burden and mild cognitive impairment and dementia: a population-based study. Brain Behav Immun. 2024;121:155–64. 39043350 10.1016/j.bbi.2024.07.026

[40] Lu Y, Liu BP, Tan CT, Pan F, Larbi A, Ng TP. Lifetime pathogen burden, inflammatory markers, and depression in community-dwelling older adults. Brain Behav Immun. 2022;102:124–34. 35202734 10.1016/j.bbi.2022.02.020

[41] Koyama T, Kuriyama N, Ozaki E, Matsui D, Watanabe I, Miyatani F, et al. Serum albumin to globulin ratio is related to cognitive decline via reflection of homeostasis: a nested case-control study. BMC Neurol. 2016;16(1):253. 27931194 10.1186/s12883-016-0776-z PMC5146886

[42] Kountouras J, Tsolaki M, Gavalas E, Boziki M, Zavos C, Karatzoglou P, et al. Relationship between *Helicobacter pylori* infection and Alzheimer disease. Neurology. 2006;66(6):938–40. 16567719 10.1212/01.wnl.0000203644.68059.5f

[43] Huang WS, Yang TY, Shen WC, Lin CL, Lin MC, Kao CH. Association between *Helicobacter pylori* infection and dementia. J Clin Neurosci. 2014;21(8):1355–8. 24629396 10.1016/j.jocn.2013.11.018

[44] Han ML, Chen JH, Tsai MK, Liou JM, Chiou JM, Chiu MJ, et al. Association between *Helicobacter pylori* infection and cognitive impairment in the elderly. J Formos Med Assoc. 2018;117(11):994–1002. 29175144 10.1016/j.jfma.2017.11.005

[45] Cárdenas V, Boller F, Román G. *Helicobacter pylori*, vascular risk factors and cognition in U.S. older adults. Brain Sci. 2019;9(12):370. 31842501 10.3390/brainsci9120370 PMC6955675

[46] Bu X-L, Yao X-Q, Jiao S-S, Zeng F, Liu Y-H, Xiang Y, et al. A study on the association between infectious burden and Alzheimer’s disease. Eur J Neurol. 2015;22(12):1519–25. 24910016 10.1111/ene.12477

[47] Beydoun MA, Beydoun HA, Elbejjani M, Dore GA, Zonderman AB. *Helicobacter**pylori* seropositivity and its association with incident all-cause and Alzheimer’s disease dementia in large national surveys. Alzheimers Dement. 2018;14(9):1148–58. 30201100 10.1016/j.jalz.2018.04.009 PMC6196325

[48] Beydoun MA, Beydoun HA, Weiss J, Hossain S, El-Hajj ZW, Zonderman AB. *Helicobacter**pylori*, periodontal pathogens, and their interactive association with incident all-cause and Alzheimer’s disease dementia in a large national survey. Mol Psychiatry. 2021;26(10):6038–53. 32366948 10.1038/s41380-020-0736-2

[49] Roubaud-Baudron C, Krolak-Salmon P, Quadrio I, Mégraud F, Salles N. Impact of chronic Helicobacter pylori infection on Alzheimer’s disease: preliminary results. Neurobiol Aging. 2012;33(5):1009.e11-1009.e19. 22133280 10.1016/j.neurobiolaging.2011.10.021

[50] Yu B, Xiang L, Peppelenbosch MP, Fuhler GM. Overlapping cytokines in H. pylori infection and gastric cancer: a tandem meta-analysis. Front Immunol. 2023;14:1125658. 37006300 10.3389/fimmu.2023.1125658 PMC10050690

[51] Tran-Chi VL, Maes M, Nantachai G, Hemrungrojn S, Solmi M, Stoyanov D, et al. Cytokine dysregulation in amnestic mild cognitive impairment. Sci Rep. 2024;14(1):22486. 39341896 10.1038/s41598-024-73099-z PMC11439069

[52] Betten Å, Bylund J, Cristophe T, Boulay F, Romero A, Hellstrand K, et al. A proinflammatory peptide from *Helicobacter**pylori* activates monocytes to induce lymphocyte dysfunction and apoptosis. J Clin Invest. 2001;108(8):1221–8. 11602630 10.1172/JCI13430 PMC209532

[53] Wang XL, Zeng J, Yang Y, Xiong Y, Zhang ZH, Qiu M, et al. *Helicobacter**pylori* filtrate induces Alzheimer-like tau hyperphosphorylation by activating glycogen synthase kinase-3β. J Alzheimers Dis. 2014;43(1):153–65. 10.3233/JAD-140198 25079798

[54] Tamura A, Fujioka T, Nasu M. Relation of *Helicobacter**pylori* infection to plasma vitamin B12, folic acid, and homocysteine levels in patients who underwent diagnostic coronary arteriography. Am J Gastroenterol. 2002;97(4):861–6. 12003420 10.1111/j.1572-0241.2002.05601.x

[55] Buzás GM. Metabolic consequences of *Helicobacter**pylori* infection and eradication. World J Gastroenterol. 2014;20(18):5226. 24833852 10.3748/wjg.v20.i18.5226 PMC4017037

[56] Berrett AN, Gale SD, Erickson LD, Brown BL, Hedges DW. Helicobacter pylori moderates the association between 5-MTHF concentration and cognitive function in older adults. PLOS ONE. 2018;13(1):e0190475. 29364915 10.1371/journal.pone.0190475 PMC5783346

[57] Chang YP, Chiu GF, Kuo FC, Lai CL, Yang YH, Hu HM, et al. Eradication of *Helicobacter**pylori* Is associated with the progression of Dementia: a population-based study. Gastroenterol Res Pract. 2013;2013:1–5. 10.1155/2013/175729 PMC3859120 24371435

[58] Kang DW, Lee JW, Park MY, Kim SH, Um YH, Wang SM, et al. Impact of *Helicobacter pylori* eradication on age-specific risk of incident dementia in patients with peptic ulcer disease: a nationwide population-based cohort study. Geroscience. 2024. 10.1007/s11357-024-01284-z. 39129052 10.1007/s11357-024-01284-z PMC11872846

[59] Kountouras J, Boziki M, Gavalas E, Zavos C, Grigoriadis N, Deretzi G, et al. Eradication of *Helicobacter pylori* may be beneficial in the management of Alzheimer’s disease. J Neurol. 2009;256(5):758–67. 19240960 10.1007/s00415-009-5011-z

[60] Kountouras J, Boziki M, Gavalas E, Zavos C, Deretzi G, Chatzigeorgiou S, et al. Five-year survival after *Helicobacter pylori* eradication in Alzheimer disease patients. Cogn Behav Neurol. 2010;23(3):199–204. 20829670 10.1097/WNN.0b013e3181df3034

[61] Keränen E, Rysä J, Tiihonen M, Hartikainen S, Tolppanen AM. Helicobacter pylori eradication treatments and risk of Alzheimer disease: a case–control study nested in the Finnish population. Epidemiology. 2025. 10.1097/EDE.0000000000001831. 39868701 10.1097/EDE.0000000000001831 PMC11957437

