# Supplementary material for: The association between H. pylori infection and cognitive deterioration: a systematic review and meta-analysis
Source: Eur J Med Res. 2025 Sep 16;30:846. doi: 10.1186/s40001-025-03160-8 (PMC12439406; doi:10.1186/s40001-025-03160-8)
Supplement: Supplementary file 1 — Supplementary material 1. [file 40001_2025_3160_MOESM1_ESM.docx]

**Supplementary Table 1.** Search strategies and search results

| **Database** | **Search strategy** | **Results** |
| --- | --- | --- |
| **Pubmed** | ("Dementia"[All Fields] OR "alzheimer*"[All Fields] OR "cognitive decline*"[All Fields] OR "cognitive dysfunction*"[All Fields] OR "cognitive impairment*"[All Fields] OR "mental deterioration*"[All Fields] OR "neurocognitive decline*"[All Fields] OR "neurocognitive dysfunction*"[All Fields] OR "neurocognitive impairment*"[All Fields] OR "neurocognitive deterioration*"[All Fields] OR "neurological decline*"[All Fields] OR "neurological deterioration*"[All Fields] OR "memory loss*"[All Fields] OR "memory disorder*"[All Fields] OR "memory dysfunction*"[All Fields]) AND ("Pylori"[All Fields] OR "campylobacter pylori*"[All Fields] OR "Helicobacter pylori"[All Fields] OR "h pylori"[All Fields]) | 234 |
| **Scopus** | TITLE-ABS-KEY ( ( "Dementia" OR "alzheimer*" OR "cognitive decline*" OR "cognitive dysfunction*" OR "cognitive impairment*" OR "mental deterioration*" OR "neurocognitive decline*" OR "neurocognitive dysfunction*" OR "neurocognitive impairment*" OR "neurocognitive deterioration*" OR "neurological decline*" OR "neurological deterioration*" OR "memory loss*" OR "memory disorder*" OR "memory dysfunction*" ) AND ( "Pylori" OR "campylobacter pylori*" OR "Helicobacter pylori" OR "h pylori" ) ) | 541 |
| **WOS** | TS=(("Dementia" OR "alzheimer*" OR "cognitive decline*" OR "cognitive dysfunction*" OR "cognitive impairment*" OR "mental deterioration*" OR "neurocognitive decline*" OR "neurocognitive dysfunction*" OR "neurocognitive impairment*" OR "neurocognitive deterioration*" OR "neurological decline*" OR "neurological deterioration*" OR "memory loss*" OR "memory disorder*" OR "memory dysfunction*") AND ("Pylori" OR "campylobacter pylori*" OR "Helicobacter pylori" OR "h pylori")) | 421 |
| **Cochrane Library** | ("Dementia" OR "alzheimer*" OR "cognitive decline*" OR "cognitive dysfunction*" OR "cognitive impairment*" OR "mental deterioration*" OR "neurocognitive decline*" OR "neurocognitive dysfunction*" OR "neurocognitive impairment*" OR "neurocognitive deterioration*" OR "neurological decline*" OR "neurological deterioration*" OR "memory loss*" OR "memory disorder*" OR "memory dysfunction*") AND ("Pylori" OR "campylobacter pylori*" OR "Helicobacter pylori" OR "h pylori") | 14 |
| **Embase** | ('dementia':ti,ab,kw OR 'alzheimer*':ti,ab,kw OR 'cognitive decline*':ti,ab,kw OR 'cognitive dysfunction*':ti,ab,kw OR 'cognitive impairment*':ti,ab,kw OR 'mental deterioration*':ti,ab,kw OR 'neurocognitive decline*':ti,ab,kw OR 'neurocognitive dysfunction*':ti,ab,kw OR 'neurocognitive impairment*':ti,ab,kw OR 'neurocognitive deterioration*':ti,ab,kw OR 'neurological decline*':ti,ab,kw OR 'neurological deterioration*':ti,ab,kw OR 'memory loss*':ti,ab,kw OR 'memory disorder*':ti,ab,kw OR 'memory dysfunction*':ti,ab,kw) AND ('pylori':ti,ab,kw OR 'campylobacter pylori*':ti,ab,kw OR 'helicobacter pylori':ti,ab,kw OR 'h pylori':ti,ab,kw) | 295 |

**Supplementary Table 2.** Quality assessment of Cohort studies with the NOS tool

| **Study ID** | **Selection** | | | | **Comparability** | **Outcome** | | | **Overall** |
| --- | --- | --- | --- | --- | --- | --- | --- | --- | --- |
|  | D1 | D2 | D3 | D4 |  | D5 | D6 | D7 |  |
| **Beydoun 2018** | * | * | * | * | * | * | * | * | Good |
| **FU 2024** | * | * | * | * | ** | * | * | * | Good |
| **Huang 2014** | * | * | * | * | * | * | * | * | Good |
| **Lu 2022** | * |  | * | * | ** | * | * |  | Good |
| **Shi 2024** | * |  | * | * | ** | * | * | * | Good |

D1: Is the case definition adequate/Representative of the exposed cohort?

D2: Representative of the cases/Selection of the non-exposed cohort.

D3: Selection of Controls/Ascertainment of exposure.

D4: Definition of Controls/ Demonstration that outcome of interest was not present at start of study.

D5: Ascertainment of exposure/ Assessment of outcome.

D6: Same method of ascertainment for cases and controls/ Was follow-up long enough for outcomes to occur.

D7: Non-Response rate/ Adequacy of follow up of cohorts.

**Supplementary Table 3.** Quality assessment of Case-control studies with the NOS tool

| **Study ID** | **Selection** | | | | **Comparability** | **Outcome** | | | **Overall** |
| --- | --- | --- | --- | --- | --- | --- | --- | --- | --- |
|  | D1 | D2 | D3 | D4 |  | D5 | D6 | D7 |  |
| **Bu 2015** | * | * | * | * | ** | * | * |  | Good |
| **Douros 2024** | * | * | * | * | ** | * | * |  | Good |
| **Kountouras 2006** | * |  | * | * | * | * | * |  | Good |
| **Koyama 2016** | * | * |  |  | * | * | * |  | Fair |
| **Shiota 2011** | * |  |  | * | * |  | * |  | Fair |
| **Tsolaki 2015** | * | * | * | * | * | * | * |  | Good |

D1: Adequate case definition/Representativeness of the cases.

D2: Selection of controls (community-based or hospital-based).

D3: Definition of controls (ensuring they are free from the outcome of interest).

D4: Ascertainment of exposure (structured interview, medical records, etc.).

D5: Ascertainment of exposure uses the same method for cases and controls.

D6: Same method of ascertainment for cases and controls.

D7: Non-response rate (ensuring missing data does not bias results).

**Supplementary Table 4.** Quality assessment of Cross-sectional studies with the NOS tool

| **Study ID** | **Selection** | | | | **Comparability** | **Outcome** | | **Overall** |
| --- | --- | --- | --- | --- | --- | --- | --- | --- |
|  | D1 | D2 | D3 | D4 |  | D5 | D6 |  |
| **Beydoun 2020** | * | * |  | * | * | * | * | Good |
| **Cardenas 2019** | * | * | * | * | * | * | * | Good |
| **Han 2017** | * | * |  | * | * | * | * | Good |
| **Roubaud-Baurdon 2012** | * |  |  | ** | * | * | * | Fair |
| **Rezaeimehr 2016** | * | * |  | * | * | * | * | Good |

**Table: Quality assessment of cross-sectional studies with NOS tool**

D1: Representativeness of the sample (Does the sample reflect the general population or target group?)

D2: Sample size (Is the study adequately powered to detect significant associations?)

D3: Non-respondents (Were non-respondents adequately described and handled?)

D4: Ascertainment of exposure (How was exposure measured? Structured questionnaire, medical records, etc.?)

D5: Outcome assessment (Was the outcome measured objectively? MRI, clinical diagnosis, etc.?)

D6: Statistical tests (Were appropriate statistical methods used to analyze the results?)
